# Shaofu Zhuyu Decoction Regresses Endometriotic Lesions in a Rat Model

**DOI:** 10.1155/2018/3927096

**Published:** 2018-01-30

**Authors:** Guanghui Zhu, Chunhua Jiang, Xin Yan, Shu Zhao, Dingjie Xu, Ying Cao

**Affiliations:** ^1^College of Traditional Chinese Medicine, North China University of Science and Technology, No. 21 Bohai Road, Caofeidian Xincheng, Tangshan, Hebei 063210, China; ^2^Guang'anmen Hospital, China Academy of Chinese Medical Sciences, No. 5 Beixiange, Xicheng District, Beijing, China

## Abstract

The current therapies for endometriosis are restricted by various side effects and treatment outcome has been less than satisfactory. Shaofu Zhuyu Decoction (SZD), a classic traditional Chinese medicinal (TCM) prescription for dysmenorrhea, has been widely used in clinical practice by TCM doctors to relieve symptoms of endometriosis. The present study aimed to investigate the effects of SZD on a rat model of endometriosis. Forty-eight female Sprague-Dawley rats with regular estrous cycles went through autotransplantation operation to establish endometriosis model. Then 38 rats with successful ectopic implants were randomized into two groups: vehicle- and SZD-treated groups. The latter were administered SZD through oral gavage for 4 weeks. By the end of the treatment period, the volume of the endometriotic lesions was measured, the histopathological properties of the ectopic endometrium were evaluated, and levels of proliferating cell nuclear antigen (PCNA), CD34, and hypoxia inducible factor- (HIF-) 1*α* in the ectopic endometrium were detected with immunohistochemistry. Furthermore, apoptosis was assessed using the terminal deoxynucleotidyl transferase (TdT) deoxyuridine 5′-triphosphate (dUTP) nick-end labeling (TUNEL) assay. In this study, SZD significantly reduced the size of ectopic lesions in rats with endometriosis, inhibited cell proliferation, increased cell apoptosis, and reduced microvessel density and HIF-1*α* expression. It suggested that SZD could be an effective therapy for the treatment and prevention of endometriosis recurrence.

## 1. Introduction

Endometriosis is a hormone-dependent benign disease that is characterized by the implantation and growth of endometrium-like glandular and stromal cells outside the uterine cavity, often associated with pelvic pain, chronic inflammation, and infertility. The reported prevalence of endometriosis is 6–10% in the general population, while it reaches up to 50% in women with infertility [1]. However, since surgical confirmation is a prerequisite for the diagnosis, the actual prevalence of the disease is probably underestimated. The pathophysiology of endometriosis is still uncertain, which poses a major obstacle in the search for a definitive treatment. Currently, laparoscopic surgery and pharmacological treatment are the standard therapeutic options for endometriosis. These treatments aim to relieve pain, optimize fertility, and delay recurrence [2]. Pharmacological treatments for endometriosis include nonsteroid anti-inflammatory drugs (NSAIDs) and hormonally active drugs such as combined oral contraceptive pills, gestrinone, danazol, and gonadotropin-releasing hormone agonists (GnRH-a). NSAIDs are used universally in women with dysmenorrhea, but there is currently no definite evidence supporting their efficacy in relieving endometriosis-induced dysmenorrhea [3].

Hormonal drugs induce hypoestrogenic state and bear considerable side effects, which inevitably counteract infertility treatment and lead to unwanted menopausal-like side effects and bone loss [4]. These side effects limit the long-term use of this type of drugs. Also the other shortcomings of hormonal therapy are the high recurrence of symptoms after discontinuation of the drugs [5]. Conservative laparoscopic surgery is a conventional treatment for patients who respond poorly to pharmacological treatment in pain relief and/or the consequential infertility. Unfortunately it was reported that 40% to 50% of patients experienced disease recurrence by 5 years after surgery [6]. Given the substantial drawbacks of current endometriosis treatment, there is enormous need for the development of new endometriosis medications that can effectively relieve the associated pelvic pain and yet possess acceptable side effect profiles; therefore they could be safely used for long-term treatment without inhibiting ovulation.

Traditional Chinese medicine (TCM) has been used to prevent and treat various diseases in China for thousands of years. TCM is on the rise worldwide with increasing popularity. It was widely used in treating major diseases and had yielded unique and satisfactory clinical outcomes [7, 8]. TCM has significant advantages in treating gynecological disorders such as endometriosis, chronic pelvic pain, abnormal uterine bleeding, dysmenorrhea, and infertility [9]. In China, TCM is commonly used for relieving endometriosis symptoms. An randomized clinical trial showed that Chinese herbal medicines prevented the recurrence of endometriosis after a conservative operation and increased the conception rate, with fewer and lighter adverse reactions as compared to GnRH-a or gestrinone treatment [10]. Similarly, a systematic review found that postsurgical administration of Chinese herbal medicine might have comparable benefits as to gestrinone or danazol treatment but was associated with fewer side effects [11]. Unfortunately, research studies on the role of TCM in treating endometriosis have been limited. Therefore, accessing the efficacy of TCM formulations and unveiling their potential mechanisms in the treatment of endometriosis are necessary and could promote the development of new treatment strategies for endometriosis.

In the theory of TCM, the etiology of endometriosis is associated with blood stasis, which is mainly caused by pathogenic cold accumulation. Dysmenorrhea, the most common symptom in patients with endometriosis, is relieved by herbal prescriptions through warming meridian channel and dissolving blood stasis as per TCM theory. The Shaofu Zhuyu Decoction (SZD) formula, originally recorded in the “Correction of Errors in Medical Classics,” a monograph compiled by Qingren Wang in the Qing dynasty, is considered a classic and valid prescription for treating cold-stagnation and blood-stasis induced dysmenorrhea. It consists of the following ten commonly used herbs: Foeniculi Fructus, Zingiberis Rhizoma, Corydalis Rhizoma, Myrrha, Rhizoma Chuanxiong, Angelicae Sinensis Radix, Radix Paeoniae Rubra, Cortex Cinnamomi, Typhae Pollen, and Trogopteri Feces, and it has been widely used in the treatment of endometriosis in clinical practice in not only mainland China, but also other districts and countries. For instance, one study that analyzed Chinese herbal medicine prescription database in Taiwan showed that SZD was one of the top five commonly used herbal formulas for the treatment of endometriosis in Taiwan [12].

The present study aimed to evaluate the effects of SZD in an endometriosis rat model and to provide further evidences of its therapeutic effect on endometriosis. We compared the volume of ectopic explants, level of cell proliferation, and apoptosis, as well as CD-34 and hypoxia inducible factor- (HIF-) 1*α* expression in ectopic lesions between SZD- and vehicle-treated rats, who had uterine tissue transplanted into abdominal cavity through surgery, using immunohistochemical method.

## 2. Materials and Methods

### 2.1. Animals

Fifty adult female Sprague-Dawley rats (weighing 180–220 g) were purchased from the laboratory animal center of Academy of Military Medical Sciences (Beijing, China). The animal experimental protocols used in this study were reviewed and approved by the Animal Care Welfare Committee of North China University of Science and Technology. All rats were allowed 1 week of acclimation to the environment and were fed ad libitum with laboratory chow and water.

### 2.2. Formula

All the herbal constituents of the formula were provided by Tangshan Branch of the Beijing Tong Ren Tang Company Ltd. The origins, medicinal parts, and weight ratios of these herbs are listed in Table 1. All herbs except cortex cinnamomi were soaked in double-distilled water for 1 h and then decocted for 1 h. The cortex cinnamomi was added last, followed by decocting for 20 min. Then the decoction was concentrated by heating. The final drug concentration was 1 g/mL.

### 2.3. Experimental Design and Treatment

After 1 week of acclimation, vaginal smears were obtained from the rats to monitor the estrous cycle. Endometriosis was surgically induced in 48 rats who had gone through two consecutive regular estrous cycles via autotransplantation, a method originally described by Vernon and Wilson [13] and later modified by Chu et al. [14]. All the rats were administered 0.2 mg estradiol valerate 24 h before surgery. At the time of surgery, the rats were anesthetized by intraperitoneal administration of 40 mg/kg pentobarbital sodium. Their abdominal skin was shaved and sterilized with 75% medicinal alcohol, and then a 2.5 cm ventral midline incision was made to expose the uterus. The right uterine horn was ligated, removed, and then placed in sterile phosphate-buffered saline (PBS). The uterine tissue was pruned to 5 × 5 mm squares with myometrium preserved. The tissue squares were then transplanted into the inner surface of the left abdominal wall adjacent to the large vessels with the endometrial surface apposed. The tissues were oversewn using a single nonabsorbable 5-0 polypropylene suture. The midline abdominal and skin incisions were then closed. After the operation, penicillin sodium was injected for 3 days and on day 10 after the surgery, all the rats were administered 0.02 mg estradiol valerate daily for 5 days.

**Figure 1:**
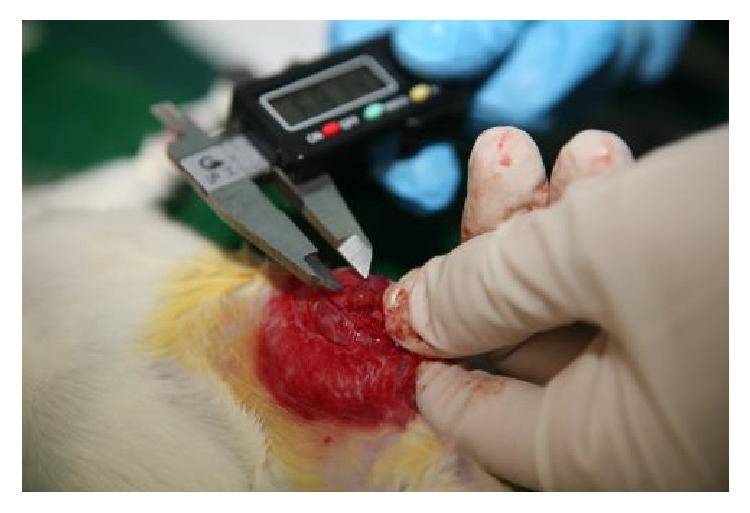
Measurement of explant volumes (length, width, and height) using digital millimetric caliper at second and third laparotomies.

At 4 weeks after surgery, all the rats underwent a second laparotomy during estrus to determine if the model of experimental endometriosis was successfully established. The length, width, and height of the explants were measured using a digital millimetric caliper and recorded (Figure 1). Based on the data collected from the second laparotomy, 38 rats were confirmed with endometriosis. All the 38 rats were injected with penicillin sodium for 3 days and were then randomly divided into two groups: SZD-treated group and vehicle-treated group. The SZD-treated rats were administered approximately 1 mL SZD daily via oral gavage daily for 4 weeks. The dose was calculated using a formula that translates doses between different species based on the animals' body surface area [15]. The body surface area was recalculated weekly based on body weight alteration to modify the administered dose. The rats in the vehicle-treated group received equal volume of normal saline daily for 4 weeks as well. After treatment period ended, all the rats were anesthetized, and a third laparotomy was performed during estrus. During the surgery, the dimensions of the implants were measured again. Then the ectopic lesions were excised, fixed in 10% formalin, and embedded in paraffin for pathological examination and immunohistochemical staining.

### 2.4. Volume Analysis

The volume of each explant was calculated using the following prolate ellipsoid formula after the second and third laparotomies: *V* (mm^3^) = 0.52 × length × width × height [16].

### 2.5. Histology

The explant tissues were cut into 4 *μ*m thick sections, followed by hematoxylin and eosin (H&E) staining. They were examined for the presence of endometriotic glands and stromal cells by a pathologist who was blinded to the group treatments.

### 2.6. Immunohistochemical Staining

Mouse monoclonal anti-proliferating cell nuclear antigen (PCNA) antibody (1:50, 610664, BD, USA) was used to detect the proliferating cells. Rabbit monoclonal anti-CD34 antibody (1:2500, ab81289, Abcam, USA) was used to evaluate the microvessel density. Rabbit polyclonal anti-HIF-1*α* antibody (1:100, ab463, Abcam, USA) was used to estimate the hypoxic status in the ectopic lesions. The sections were deparaffinized and rehydrated using xylene and ethanol washes. Then the endogenous peroxidase activity was inactivated by incubation with 0.3% hydrogen peroxidase for 20 min and antigen retrieval was performed by boiling the sections in 0.01 M sodium citrate buffer for 30 s, followed by cooling to room temperature. After incubating in goat serum for 30 min to block the nonspecific antibodies, the tissues were incubated with primary antibody overnight at 4°C.

After washing three times with PBS, the sections were incubated with the secondary antibody (PV-900, Zhongshan Jinqiao, China) for 30 min at 37°C. Subsequently, the sections were washed again in PBS, incubated with 0.01% 3,3-diaminobenzidine tetrahydrochloride (DAB) for 2 min, and counterstained with hematoxylin for 20 s. Finally, the sections were rinsed with water and dehydrated with alcohol and xylene. On each section five fields were selected randomly under a high power field using a BX53 microscope (Olympus). The Image-pro plus 6.0 (IPP) software was used for assessing the ratio of PCNA- and HIF-1*α*-positive cells and the microvessel density.

### 2.7. Terminal Deoxynucleotidyl Transferase (TdT) Deoxyuridine 5′-Triphosphate (dUTP) Nick-End Labeling (TUNEL)

Cell apoptosis was evaluated using the ApopTag peroxidase in situ apoptosis detection kit (S7100, Chemicon, USA) according to the manufacturer instructions except that DAB was used for counterstaining instead of methyl green. The ratio of apoptotic cells was also assessed using the IPP software as described above.

### 2.8. Statistical Analysis

The statistical analysis was performed using the Statistical Package for the Social Sciences (SPSS) version 22.0, and the experimental results were presented as the mean ± standard deviation (SD). All the data were first analyzed for normal distribution and equal variance and then compared using an independent sample *t*-test or one-way analysis of variance (ANOVA). Statistical significance was accepted at *P* < 0.05.

## 3. Results

### 3.1. Treatment with SZD Attenuates Lesion Volume

Endometriotic explants were implanted in 38 rats as per the protocol. During the intervention, one rat from each group, vehicle- and SZD-treated, died, of oral gavage incident and a bite wound, respectively. The results showed that, 4 weeks after transplantation, the mean volumes of the lesions were comparable between the two groups. However, at the end of the treatment (8 weeks after transplantation), the mean volumes of the lesions were lower in the SZD-treated group than they were in the vehicle group. The mean volume of the lesions decreased from 33.57 ± 18.44 mm^3^ to 8.66 ± 11.304 mm^3^ in the SZD-treated group (*P* < 0.01). In contrast, the mean volume of the lesions increased from 32.31 ± 23.67 mm^3^ to 40.47 ± 19.60 mm^3^ in the vehicle group (*P* < 0.05, Table 2).

### 3.2. SZD Alters Cellular Organization in Ectopic Lesions

**Figure 2:**
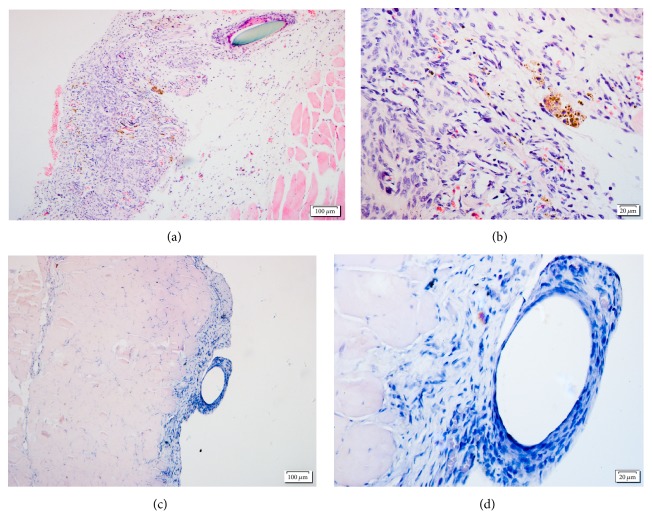
Ectopic endometrium of vehicle (a, b) and Shaofu Zhuyu Decoction (SZD) groups (c, d). Stain: hematoxylin and eosin (H&E). Scale bars (a and b) = 100 *μ*m; (c and d) = 20 *μ*m.

The ectopic lesions were processed for H&E staining. The lesions from the SZD-treated groups showed the regression of endometrial glands and glandular epithelium and numerous stromal cells while the lesion sections of vehicle group showed viable glandular tissue as well as more epithelial and stromal cells (Figure 2).

### 3.3. Treatment with SZD Inhibits Cell Proliferation and Increases Cell Apoptosis

**Figure 3:**
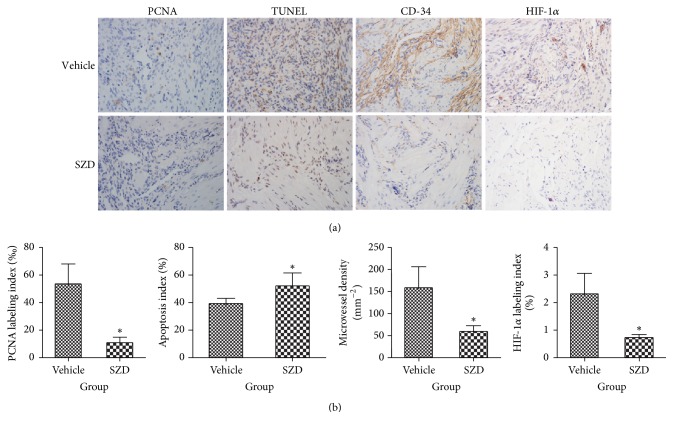
Immunohistochemical detection of makers in endometriotic lesions. (a) Representative immunohistochemical images of ectopic endometrium from vehicle- and Shaofu Zhuyu decoction- (SZD-) treated rats are shown. Sections were stained with PCNA to detect proliferating cells, TUNEL assay to confirmed apoptosis, CD-34 to detected microvessels, and HIF-1*α* to evaluate hypoxia status; magnification: ×100. (b) PCNA-positive (‰), apoptosis index (%), microvessel density (mm^−2^), and HIF-1*α*-positive (%) cells in endometriotic lesions after treatment, analyzed with quantitative analysis of immunohistochemical sections. Mean ± standard deviation (SD). SZD group (*n* = 8) versus vehicle group (*n* = 7): ^*∗*^*P* < 0.01.

Cell proliferation was evaluated using immunohistochemical staining of PCNA (Figure 3). The semiquantitative analyses showed less PCNA staining cells in the SZD-treated rats as compared to in the vehicle group rats. In addition, cell apoptosis was evaluated using TUNEL assay (Figure 3). The number of TUNEL-labeled cells in the SZD-treated rats was higher than that in the vehicle group.

### 3.4. SZD Reduces Microvessel Density and HIF-1*α* Expression

The effects of SZD on microvessel density and HIF-1*α* expression were assessed using immunohistochemical staining (Figure 3). Compared with the vehicle group, the average microvessel density and HIF-1*α* expression were significantly lower in the SZD-treated group.

## 4. Discussion

Since endometriosis is considered as an estrogen-dependent disease, most of the current medications aim to reduce estrogen levels. However, the notable side effects of estrogen reduction have limited the long-term use of such drugs. Currently, the development of endometriosis is believed to be associated with multiple factors such as steroid metabolism, inflammation, immune system abnormalities, and genetic changes [17, 18]. Nevertheless, the definite mechanisms underlying the development of endometriosis are far from clear, which challenges the development of a proper curative agent. TCM, a medicine practiced clinically for thousands of years, recognizes diseases based on the holistic concept. It is characterized by personalized therapeutic formulation in the treatment of diseases, which highlights the difference from Western medicine.

Adopting TCM knowledge into the clinical practice would facilitate the development of a more effective strategy for treating diseases whose mechanisms remain unclear to the Western medicine professionals, such as endometriosis. Notably the 2015 Nobel Prize in Physiology or Medicine encouraged the Western medical community to recognize the importance of TCM. SZD as a complex prescription is a classic prescription for treating dysmenorrhea and has been used in the last two centuries for this indication. It is widely used to treat endometriosis currently [12]. The results of this study showed that after a 4-week treatment period, SZD attenuated the size of ectopic lesions, which provides evidence to support the consideration of using SZD clinically as an effective medication for endometriosis.

Cell proliferation and apoptosis, the two basic cell behaviors, play a pivotal role in pathogenesis of ectopic lesions and determine the progression and regression of lesions. Numerous* in vivo* and* in vitro* studies have demonstrated that hormones, oxidative stress, and proinflammatory cytokines regulate cell proliferation and apoptosis by activating nuclear factor-kappa B or the mitogen-activated protein kinase (MAPK) and extracellular signal-regulated kinase (ERK) 1/2 [19–21]. Although the mechanisms regulating cell proliferation and apoptosis have not been fully elucidated, the inhibition of cell proliferation and induction of apoptosis are still believed to be promising treatment strategies. GnRH-a as a classic hormonally active drug for endometriosis increases the apoptotic cell index and decreases cell proliferation and therefore facilitates the regression of ectopic lesions at the tissue level [22, 23]. Some emerging agents, such as honokiol [24], resveratrol [25], sunitinib [26], imatinib [27], and melatonin [28], are believed to be potential medications for endometriosis treatment. They reduce the size of ectopic lesions in animal models of endometriosis by inhibiting cell proliferation or inducing apoptosis or both. According to the results of the immunohistochemical staining and TUNEL assay in this study, SZD was shown to decrease cell proliferation and increase apoptosis of explants in endometriosis-induced rats. Similarly, studies on Guizhi Fuling Capsule [29] and Guixiong Xiaoyi Wan [30] also reported that the investigational drug attenuated the size of explants in rats with endometriosis through the induction of apoptosis.

Ample evidence suggests that angiogenesis is pivotal in the establishment and progression of the endometriotic lesion, and therefore antiangiogenesis is widely accepted as an emerging potential target for endometriosis treatment [31–33]. CD-34, a marker for small vessel endothelium, was used to assess the microvessel density. The results of this study suggested that SZD could decrease the MVD within endometriotic lesion significantly. HIF-1*α*, as a transcription factor that could regulate the expression of hundreds of genes under hypoxic conditions, was reported to play an important role in the progression of endometriosis in several studies. For example, HIF-1*α* could promote endometrial stromal cells migration and invasion [34], induce epithelial-mesenchymal transition of endometrial epithelial cells [35], and upregulate vascular endothelial growth factor expression [36, 37]. In addition, inhibiting the expression of HIF-1*α* suppressed the growth of lesion in animal models with endometriosis [38, 39]. Our study proved that SZD reduces the expression of HIF-1*α*, which might have contributed to the reduced size of the ectopic lesions.

The ectopic endometria from patients with endometriosis were reported to exhibit a higher proliferation and antiapoptosis as well as elevated expression of HIF-1*α* [40]. These attributes could have contributed to the survival of ectopic endometrial cells under hypoxia conditions at the early stage when endometrium debris retrogrades into the pelvic cavity. Since dynamic angiogenesis occurs in early endometriotic lesions, the antiangiogenesis agents are likely to be beneficial for early-stage disease or to prevent recurrence after surgery [41]. Our study suggested that SZD could reduce cell proliferation, increase cell apoptosis, and inhibit angiogenesis and HIF-1*α* expression. Therefore, it appears that SZD may be beneficial for preventing the recurrence of endometriosis after surgery. However, further studies are needed to reveal whether SZD delay or reduce recurrence. In addition, as most patients after surgery desire a successful pregnancy, studies that investigate if SZD increase the conception rate for the said patient population are warranted too.

## Figures and Tables

**Table 1:** Composition of Shaofu Zhuyu Decoction (SZD).

Latin name	Chinese name	Medicinal parts	Origin	Grams	Weight ratio
Foeniculi Fructus	Xiaohuixiang	Seed	Shanxi, China	3	6.6
Zingiberis Rhizoma	Ganjiang	Root	Sichuan, China	0.6	1.3
Corydalis Rhizoma	Cuyanhusuo	Tuber	Zhejiang, China	3	6.6
Myrrha	Moyao	Resin	Fujian, China	3	6.6
Chuanxiong Rhizoma	Chuanxiong	Root	Sichuan, China	3	6.6
Angelica Sinensis Radix	Danggui	Root	Gansu, China	9	19.7
Radix Paeoniae Rubra	Chishao	Root	Neimenggu, China	6	13.2
Cortex Cinnamomi	Rougui	Cortex	Guangxi, China	3	6.6
Typhae Pollen	Puhuang	Pollen	Hebei, China	9	19.7
Trogopteri Feces	Wulingzhi	Feces	Hebei, China	6	13.2
Total amount				45.6	100

**Table 2:** Volume of lesions before and after treatment.

Group	Pretreatment (mm^3^)	Posttreatment (mm^3^)
Vehicle (*n* = 18)	32.31 ± 23.67	40.47 ± 19.60^*∗*^
SZD (*n* = 18)	33.57 ± 18.44	8.66 ± 11.304^△^

*Note*. ^*∗*^*P* < 0.05 and ^△^*P* < 0.01 compared with pretreatment.
